# A Domain-Independent Generative Adversarial Network for Activity Recognition Using WiFi CSI Data

**DOI:** 10.3390/s21237852

**Published:** 2021-11-25

**Authors:** Augustinas Zinys, Bram van Berlo, Nirvana Meratnia

**Affiliations:** Interconnected Resource-Aware Intelligent Systems Cluster, Department of Mathematics and Computer Science, Eindhoven University of Technology, 5600 MB Eindhoven, The Netherlands; b.r.d.v.berlo@tue.nl (B.v.B.); n.meratnia@tue.nl (N.M.)

**Keywords:** device-free sensing, unobtrusive sensing, WiFi CSI, generative adversarial network, domain change, domain adaptation

## Abstract

Over the past years, device-free sensing has received considerable attention due to its unobtrusiveness. In this regard, context recognition using WiFi Channel State Information (CSI) data has gained popularity, and various techniques have been proposed that combine unobtrusive sensing and deep learning to accurately detect various contexts ranging from human activities to gestures. However, research has shown that the performance of these techniques significantly degrades due to change in various factors including sensing environment, data collection configuration, diversity of target subjects, and target learning task (e.g., activities, gestures, emotions, vital signs). This problem, generally known as the domain change problem, is typically addressed by collecting more data and learning the data distribution that covers multiple factors impacting the performance. However, activity recognition data collection is a very labor-intensive and time consuming task, and there are too many known and unknown factors impacting WiFi CSI signals. In this paper, we propose a domain-independent generative adversarial network for WiFi CSI based activity recognition in combination with a simplified data pre-processing module. Our evaluation results show superiority of our proposed approach compared to the state of the art in terms of increased robustness against domain change, higher accuracy of activity recognition, and reduced model complexity.

## 1. Introduction

With the accelerating development of new sensing and communication technologies, monitoring human activities in everyday life has become more popular than ever in various fields such as surveillance, entertainment, and healthcare. Sensing technologies in the field of human context (e.g., activities, gestures, emotions, vital signs) recognition can be categorised into two sub-categories: device-based and device-free. While device-based sensing refers to a situation in which sensors are attached to the human body to measure and monitor a specific context, device-free sensing refers to situations in which not the human body, but the environment in which a human is present in is monitored.

Although many device-based sensing systems have become quite popular, in some situations it is impractical and cumbersome to wear them all the time. In order to overcome the limitations of device-based sensing, device-free sensing, such as visual-based sensing (cameras), has been considered. Although this technology is quite popular, with computer vision algorithms (object detection/recognition) advancing rapidly, it only operates in scenarios in which a subject is in line-of sight and no occluding obstacles are in the view. Additionally, it requires robust and continuous lighting conditions, as a subject may not be visible throughout the entire day. Moreover, visual-based sensing devices are intrusive as they impact the privacy of an individual. Therefore, in order to overcome all of these limitations, device-free solutions, using radio signals such as WiFi, are considered. In particular, the IEEE 802.11 protocol contains channel state information (CSI), which characterizes how well wireless signals propagate from a transmitter to a receiver at a certain carrier frequency [1]. WiFi CSI contains carrier signal amplitude and phase. Recently, it has been commonly used for fine-grained activity recognition in combination with data-driven, learning-based models [2,3,4,5,6,7,8].

However, research has shown that, the performance of these learning-based approaches in the context of WiFi CSI-based activity recognition significantly degrades due to various domain factor changes. For instance, it was shown that performance of WiFi-based activity recognition systems are impacted by a change of the environment in which the activity is performed [5,7,9], change of orientation with respect to the sensing device [5], CSI data quality [1], different physical properties of human subjects or slight difference in movement patterns of a user [4,6]. Even the time of day may have a big impact, as electromagnetic waves are impacted differently during the day and night in office or home environments [4,10]. Therefore, the focus of this paper is on addressing the performance degradation issue discussed above, which is referred to as “domain change”.

Typically, the domain change problem can be addressed by collecting more data and learning the data distribution that covers multiple factors impacting the performance. However, activity recognition data collection is a very labor-intensive and time consuming task, and yet there are too many known and unknown factors impacting WiFi CSI signals. In particular, each new environment setup has multiple paths from the transmitter to receiver [9]. Therefore, a learning-based system trained once on one particular environment, activity, or human subject at a specific time may not be sufficiently robust and consistent against the change. This indicates the need for robust recognition models, which are capable of performing well independent of the factors that a recognition system is exposed to.

### Contributions

The main contributions of this paper are:

- A new generative adversarial network for domain adaptation with UNet architecture and a simplified set of CSI pre-processing module.
- A convolutional neural network model combined with a triplet loss for feature extraction.
- A thorough impact analysis of various internal parameters and design choices of the proposed generative adversarial network and convolutional neural network models.

## 2. Problem Statement

In order to formalize the “domain change” problem, we present it using clear mathematical notations. First of all, a WiFi-based activity recognition system operates in domain $D(a_{1},a_{2},\ldots ,a_{n})$, which contains *n* number of domain attributes $a_{n}$ that impact performance either negatively or positively. In general, for a given domain, denoted by D, an activity recognition task, denoted by T, can be written as a set {Y,P(Y|X)}, where *P* is a function of a conditional probabilistic model, *Y* is a label space and X∈D is the input data space. Using supervised learning techniques, P(Y|X) is learnt from the labeled data $\left\{x_{i},y_{i}\right\}$, where $x_{i}\in X$ and $y_{i}\in Y$.

Let us assume that the source domain, denoted by $D^{s}$, is used for training, the target domain, denoted by $D^{t}$, is used for testing, and a recognition task is defined as $T^{s}=\left\{Y^{s},P\left(Y^{s}|X^{s}\right)\right\}$ and $T^{t}=\left\{Y^{t},P\left(Y^{t}|X^{t}\right)\right\}$. In normal cases, i.e., if $D^{s}=D^{t}$ and $T^{s}=T^{t}$ [4,11,12], the learning-based model of activity recognition there will have no performance degradation. In case of domain change, however, the tasks do not change (which means $T^{s}=T^{t}$), but the source and target domains are no longer the same (which means $D^{s}\neq D^{t}$) [11], resulting in activity recognition performance degradation.

The domain shift problem can be further categorized into two categories: (i) homogeneous, where the input data space of the domain attributes $a_{n}$ are the same (which means $X^{s}=X^{t}$), but the data distribution is not (which means $P{\left(X^{s}\right)}^{s}\neq P{\left(X^{t}\right)}^{t}$) and (ii) heterogeneous, where the input data space is different $X^{s}\neq X^{t}$ [11,13]. In the former case, there is an assumption that domains differ only in marginal distributions. Therefore, domains can be adapted by correcting the sample selection bias. The latter case, however, is more challenging, as the input data space of $a_{n}$ is available from the source domain, but it is represented in a different way than that of the target [13]. In order to relate the general domain change problem to the context of WiFi CSI-based activity recognition, both homogeneous and heterogeneous cases will be discussed, with the focus on major body activities such as various hand gestures or body movement/exercises, as described in Section 5.1 further explored in this paper.

Regarding the homogeneous case, marginal probability distributions data spaces are different ($P{\left(X^{s}\right)}^{s}\neq P{\left(X^{t}\right)}^{t}$). For instance, they are different if a recognition system is trained to classify major hand movements in an environment/room, where electromagnetic wave interference is lower and less frequent (suburbs) than in the target domain (city). Moreover, probability distributions may differ, for example, when a group of people (source domain) performs specific body activities less often than another group of people (target domain). The performance of a learning model may degrade in both examples as the marginal probability distributions between the source and target domains (environments and people respectively) are different. Regarding the heterogeneous case, the input data space in the source domain is different compared to the target domain ($X^{s}\neq X^{t}$). For instance, assuming a learning model is trained on one group of people—female (source domain)—with different physical properties $a_{n}$ than the other group of people—male (target domain)—then the way that the movements of the activities are performed would be different. Consequently, this would cause a domain change problem, as the input data space of $a_{n}$ (physical body properties) between groups of female and male are not the same. Additionally, physical properties may be different regionally based on the average human height or any other physical property that is relevant to one specific geographical region or human race, etc.

## 3. Related Work

Different methods have been developed in the past for domain independent activity recognition using WiFi CSI data. These methods can be categorized into two main categories, i.e., (i) model-based, and (ii) learning-based [10]. Since most of the learning-based approaches have shown huge success in the recent years, model-based approaches will not be reviewed in this paper. Learning-based approaches are data-driven and can be trained to perform an activity recognition task using a machine learning algorithm. These learning-based approaches may be categorized into three sub-categories, i.e., supervised, semi-supervised, and unsupervised learning [14]. Based on this categorization, we present in the following sections an overview of various deep learning methods, designed for activity recognition using WiFi CSI data.

### 3.1. Supervised Learning Approaches

Various supervised learning algorithms have been successfully applied for activity recognition using WiFi CSI data. Supervised learning algorithms are based on training with labelled data sets, where each piece of input data *x* has an associated label *y* and a model can learn to extract relevant features to map each input *x* to a corresponding output label *y*. This algorithm training setting is quite common in the literature.

A baseline paper, called SignFi [6], was published in 2018. Authors designed a deep learning model based on convolutional neural networks (CNN) to recognize hand sign gestures. In total, 5 people volunteered, and CSI traces of 276 different sign gestures in two different environments, i.e., home and lab, were collected. Authors showed that the performance of classical machine learning methods, such as k-Nearest Neighbour, degrades with the increasing number of gesture categories. Although their model showed high performance in each individual environment, based on leave-one-subject-out cross validation, they showed that their model degrades on a new user that the model has not been trained on.

Authors of [15] addressed the domain change problem in the field of hand gesture recognition. Their model worked well in new environments with minimal tuning and few additional training samples. In order to achieve this, they proposed to use Siamese neural network architecture [2], where two identical twin networks were used with shared weights and two input samples were fed to each of the two networks. They first utilized the convolution neural network to extract spatial information, and then coupled it with the recurrent neural network BILSTM (Bi-directional Long-Short Term Memory) to capture temporal information. Secondly, they proposed to use a pairwise loss function with the combination of Mk-MMD (multiple kernel maximum mean discrepancies) [15]. While this pairwise loss function maximizes the L2 distance between the samples of different classes, and minimizes the distance between the gesture samples of the same class, MMD aids better domain adaptation. In order to test their architecture, they invited 10 volunteers to collect data on six major hand movements in two different meeting rooms (large and small). Overall, the authors concluded that their architecture improved existing methods under very small sample conditions, however its performance degraded with fine grained finger gestures.

Authors of [5] introduced a robust supervised cross-domain recognition system. They collected one of the largest open-source WiFi CSI datasets, which will be described in more details in Section 5.1 and will be used throughout this paper. It consists of seventeen subjects performing various hand gestures in three different environments. The dataset was used to study domain independent features, which eres found to be the body velocity profile (BVP). Authors developed a pre-processing module, which transforms CSI data to signal power distribution over velocity components in the body coordinate system. Regarding the classification, a hybrid spatial-temporal deep learning model was designed. Their model takes the BVP as an input, and outputs prediction of the gesture. Based on the experiments, their entire recognition system achieves quite robust results, being environment, person, location and orientation independent. The major contribution of this paper is the use of the BVP as an input feature.

### 3.2. Un/Semi—Supervised Learning Approaches

Unsupervised and semi-supervised learning have shown huge success, outperforming most of the supervised learning activity recognition models using WiFi CSI data. Both semi-supervised and unsupervised learning are beneficial when labeled data is not available or is too expensive or time consuming to label.

Authors of [3] achieved model robustness in different environments by utilizing the unsupervised domain adaptation [16]. They employed labeled and unlabeled parts of the dataset to train three neural networks for feature extraction, activity recognition, and domain discrimination via an adversarial learning approach. Although the proposed model uses both labeled and unlabeled data, each environment had to be labeled to get a clear distinction. Due to the fact that the model requires new discriminator construction every time additional domain factors are introduced, this may lead to scalability problem, when the system is going to be deployed and used in real life and as such needs to be addressed in the future.

Authors of [9] introduced WiADG to identify human gestures accurately under different environmental dynamics using adversarial domain adaptation [16]. Their main system architecture was divided into three steps. In the first step, authors assumed that only source domain training data is available, so they trained a source encoder and source classifier to get high performance only in one specific source environment. Then, in the second step, an unsupervised adversarial domain adaptation technique was applied, by utilizing trained source encoder, new target encoder, and an environment discriminator. Inspired by generative adversarial networks, where the discriminator distinguishes between the fake and real images, the WiADG discriminator separates the source and target domains. The main learning objective was set such that target encoder forced the discriminator to classify unlabeled target samples as source samples, while the discriminator seeks the opposite. Finally, in the last step, a trained target encoder and source classifier were used to identify gestures in the target domain. In order to test performance, authors collected CSI data on various hand gestures in two different environments, i.e., office and conference room. They showed that with adversarial domain adaptation, their system improved the overall accuracy.

#### Generative Adversarial Network-Based Approaches

Since our proposed method is based on generative adversarial networks (GANs) [17], in this section we focus on techniques inspired by GAN. A GAN-based semi-supervised architecture was proposed in [8]. The authors addressed performance degradation of leave-one-subject-out, when the data of one person was used for training (source domain) and another for testing (target domain). The main contribution of the paper is that they used two generators in their GAN architecture. One generator is a vanilla GAN [17] to generate fake samples, and another generator is a complement GAN, trained using the “CycleGAN” [18], which generates the source domain samples in accordance with the data of the left-out user (target domain). In order to test their performance, authors used SignFi [6] and Falldefi [19] datasets. Although, they had quite a low performance degradation when performing leave-one-out-subject validation, they did not test the performance of their model on data from different environments. Finally, they faced some stability issues during training.

Another paper based on GAN is WiGAN [7], in which the authors showed that their proposed modelis environmental and user independent. First, the authors combined the structure of Deep Convolutional GAN [20] with the main characteristics of Conditional GAN [21]. This combination resulted in altered generator *G* input, which consists of the prior latent *z* together with the sample label $y_{i}\in Y$. This improved sample generation, solving the small sample problem. The second contribution was the discriminator network *D*, for which they proposed a convolutional neural network module to fuse the feature maps of the last four layers of *D*. Those compressed feature maps were then further processed by the “softmax” activation function to output the probability distribution of each category, which is used in GAN training. Taking the feature maps of the last four layers instead of only the last one provided an effective way to learn by choosing an optimal set of features. The third contribution was related to the fact that, instead of using the discriminator *D* directly as a classifier, the authors proposed to use Support Vector Machine (SVM) [22]. Based on the experiments with Widar3.0 dataset [5], they showed that SVM outperforms CNN under small sample conditions. The trade-off, however, was that performance was degraded while testing on a large number of activity categories. The authors did not further explore different classification methods based on deep learning and left it for future studies.

Furthermore, the authors of [7] proposed to add a data pre-processing module for the training deep learning model, whose main purpose was to convert raw CSI data into the sanitized CSI amplitude. This module consists of several data pre-processing steps, such as activity detection, interpolation, Discrete Wavelet Transform and sub-carrier selection. Several experiments were performed to check the impact of the data pre-processing module on overall model performance. It was showed that, without it, 10% to 20% performance degradation was experienced. Although this indicates that the raw data already contains unnecessary noisy components and it should be pre-processed, the authors did not further explore which pre-processing steps are the most optimal ones and did not experiment with other methods available in the literature.

## 4. A Generative Adversarial Network Pipeline for Activity Recognition Using WiFi CSI Data

In this section, we first explain the standard pipeline of learning-based activity recognition models using WiFi CSI data and its common building blocks. We then present our contribution, including the new generative adversarial networks, CNN-based feature extraction, and simplified CSI data pre-processing.

**Figure 1:**
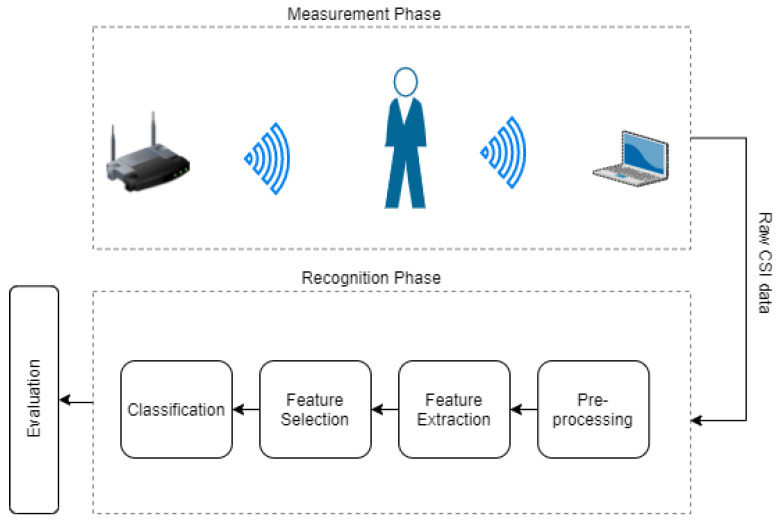
Standard pipeline of activity recognition using WiFi CSI data.

**Figure 2:**
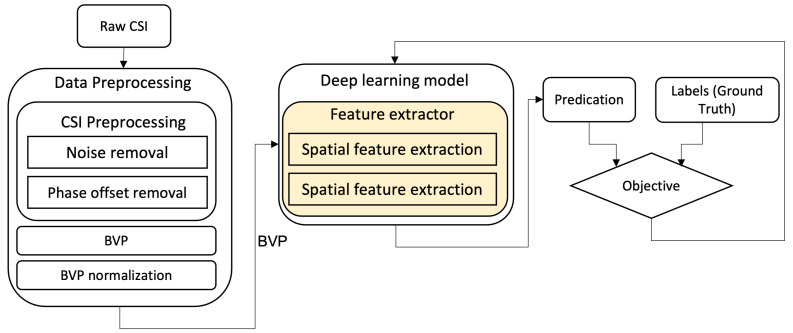
Pipeline of Widar3.0 paper’s activity recognition using WiFi CSI data, taken from [5].

The standard pipeline of activity recognition (see Figure 1) consists of building blocks for data pre-processing, feature extraction, feature selection, and classification. Recent deep learning-based activity recognition using WiFi CSI data has adapted this standard pipeline by replacing some parts with artificial neural networks. For instance, Figure 2 depicts the Widar3.0 [5] deep learning-based activity recognition pipeline, which is used as a reference for comparison in this paper. It can be seen from the figure that (i) feature extraction, feature selection, and classification are replaced by a deep learning model and (ii) the data pre-processing phase consists of several stages. Firstly, the noise and phase offsets are removed in the pre-processing phase. Then, using pre-processed CSI data, a body velocity profile (BVP) is generated. Finally, the BVP is normalized for the input of the deep learning model.

**Figure 3:**
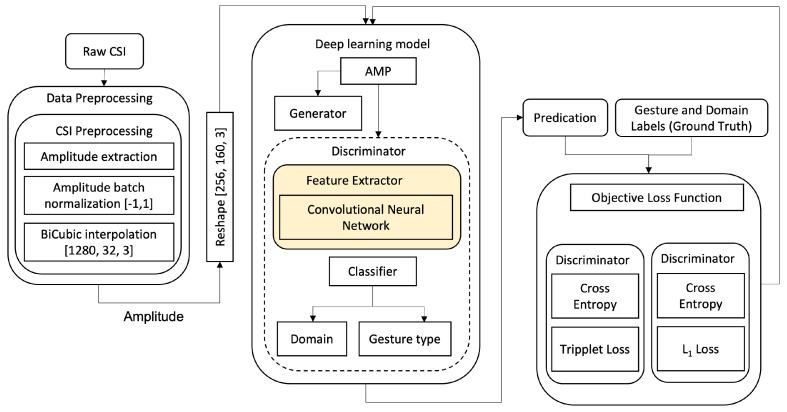
Our proposed generative adversarial network-based pipeline (ADA).

In this paper, we propose a generative adversarial network-based pipeline, illustrated in Figure 3, with a simplified pre-processing module. The adversarial training, using which the generator competes against the discriminator to classify CSI gestures and to categorise the domains such as subject ID or room ID. The generator is generating CSI gesture amplitude samples in such a way that it confuses the discriminator to classify the sample domain, leading the discriminator to a classification performance independent of the domain change.

Our data pre-processing begins with amplitude data extraction from raw CSI, followed by amplitude normalization and interpolation. The former is required due to the fact that standard neural networks do not provide support for complex numbers and for a more stable training process [23]. Research regarding deep neural networks that support complex numbers is an emerging research field [24]. Regarding the latter, amplitude sample constant shape is required for the input of the artificial neural network. Whilst there are methods to construct a CNN model with varying input shape [25,26], this is out of the scope of this paper and is left for future work. Our deep learning model, as it can be seen in Figure 3, is different than the deep learning model of the Widar3.0 paper, as it utilizes only spatial feature extraction—a Convolutional Neural Network, which directly takes normalized and interpolated CSI amplitudes as input. The reason we opt for a CNN architecture for feature extraction is that various papers have shown that deep learning approaches, in which the model learns the features by itself, can achieve higher performance compared to models that use hand-crafted/statistical features.

### 4.1. WiFi CSI Data Pre-Processing

Data pre-processing plays an important role in addressing the domain change problem in learning-based activity recognition systems using WiFi CSI data. Based on recent research papers, the modern deep learning approaches discussed in Section 3, proposed data pre-processing pipelines that increase recognition system performance by approximately 20% [7] or 40% [5]. However, as discussed in Section 3, commonly used pipelines in the literature consist of a long list of different methods, and it is quite complex to find an optimal combination of these techniques. Thus, we strive to automate the pre-processing phase by allowing the convolutional neural network to learn the optimal set on its own. With this idea, we further analyze different methods to reduce the complexity of the standard pre-processing pipeline presented in Figure 2. We aim to find minimal CSI data pre-processing operations required by the convolutional neural network to function well in the presence of domain change.

**Figure 4:**
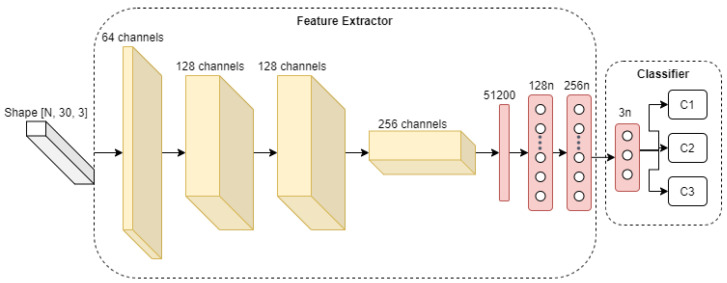
Convolutional neural network used for activity classification.

To study the effects that our choice of data pre-processing steps will have on the overall performance of the activity recognition system, we use a simple four-layer convolutional neural network, as depicted in Figure 4 for activity classification, with results presented in Section 5.2. As it can be seen, the network receives an input of pre-processed CSI amplitude. Then pre-processed sample is further convolved by four convolutional layers, with a kernel size of (3,3), stride (2,2) and the “LeakyReLU” activation (α=0.1) function. Subsequently, the last convolutional layer output (with shape [100,2,256]) is flattened to a vector of size 51,200, which is then passed to three densely connected layers, with 256, 128, and 3 neurons each. Finally, the output of the neural network is passed through a Softmax activation function to obtain the probability of each gesture category. Regarding the training process, the network weights are initialized with the Glorot uniform distribution [27] and trained using Adam [28] optimizer, with a learning rate of $10^{-3}$ and objective loss function (categorical cross-entropy).

### 4.2. A CNN-Based Architecture for Feature Extraction

**Figure 5:**
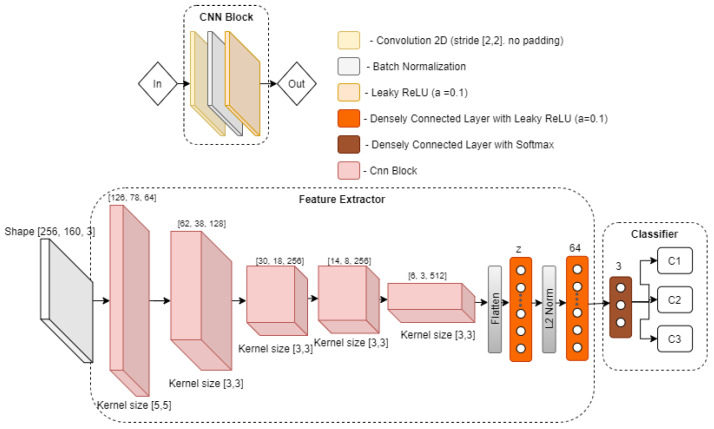
CNN feature extractor architecture.

The base architecture of the CNN model used for our feature extraction component is shown in Figure 5, which will be used and updated with each experiment in Section 5.3. The CNN feature extractor consists of five CNN blocks, where the first block takes an input of CSI amplitude in a reshaped form [256,160,3]. Each block reduces its input by approximately twice, and contains three operations, i.e., convolution, batch normalization, and LeakyReLU activation. Then convolutional part of the model is followed by a stack of four layers, which starts with an operation to flatten out feature maps of the last CNN layer, followed by a densely connected layer of size variable *z* (latent dimension), which will be varied in the experiments. After that, the vector *z* is processed with $L_{2}$ normalization, which is then passed to a densely connected layer of size 64. Finally, the output of the feature extractor is then passed to a classification module, which contains a densely connected layer, with three neurons, followed by softmax activation, in order to output the probability distribution of the three type of gestures.

### 4.3. Adversarial Network Architecture

**Figure 6:**
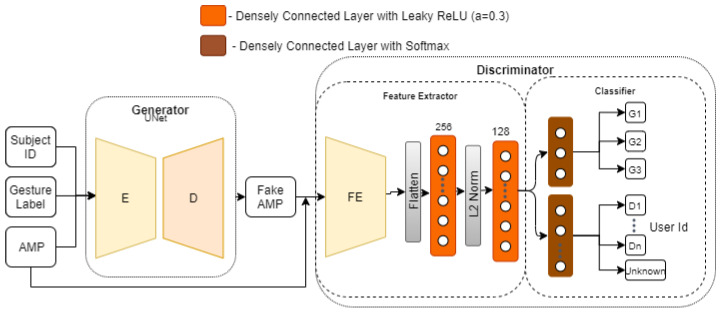
Adversarial network model.

Figure 6 shows a high-level overview of the architecture of our adversarial network. It consists of two artificial neural networks, i.e., (i) a generator and (ii) a discriminator, which compete against one another. For the discriminator part of the model, we use the final outcome of experiments discussed in Section 5.3 as our feature extractor and classifier. We then borrow the idea from [3] to discriminate between the domains and gestures using the domain discriminator (DD) and gesture discriminator (GD), respectively, in our classifier module. In our case, we use the subject ID as domain labels and leave out other possible domain labels, such as room ID, face orientation, or subject location in a room for future studies. The generator and discriminator networks are trained one after another with a data batch of size 32, an Adam optimizer (with learning rate of 0.0002 and $\beta_{1}=0.5$), until it is observed that the gesture classification test accuracy ceases to improve.

**Figure 7:**
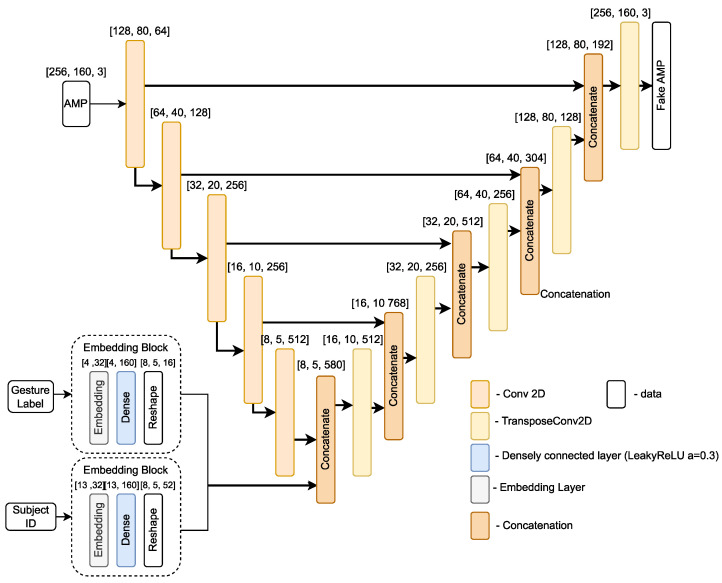
UNet model architecture.

For the generator, we use a U-Net based architecture [29], which was originally designed for translating an input image to its corresponding output image by sharing information between intermediate layers of encoder and decoder. Based on this idea, we propose a UNet based architecture variant that translates the CSI amplitude *x* into a fake amplitude $x_{f}$. The proposed architecture is depicted in Figure 7. It takes an input of pre-processed CSI amplitude sample, one hot encoded gesture label, and subject ID. Then, based on the provided input, it translates the amplitude sample *x* into a fake amplitude sample $x_{f}$, which is then passed to the discriminator.

The overall objective functions, which have to be minimized by the discriminator and the generator, are described in Equations (1) and (2), respectively. Regarding the discriminator, it outputs probability classes of three gesture categories and *k* probability classes for domain categories, where (k+1)th is for unknown. As can be seen in Equation (1), the loss $L_{D}$ consists of four terms. The first term corresponds to gesture discriminator (GD) loss with real gesture samples (*x*, $y_{g}$). The second term is the loss term for domain discriminator (DD) with real gesture samples (*x*, $y_{d}$), where $y_{d}$ corresponds to subject ID. Finally, the third and fourth terms correspond to domain discriminator (DD) loss with input of fake sample ($x_{f}$, $y_{d}$), where $y_{d}=k+1$—unknown domain category and triplet loss $L_{T}$ (margin α=1.75), respectively.

(1) $$\begin{array}{cc} L_{D}= & -E_{x,y_{g}∼p_{data}\left(x,y_{g}\right)}\log\left[p_{GD}\left(y_{g}|x\right)\right]\\ \; & -E_{x,y_{d}∼p_{data}\left(x,y_{d}\right)}\log\left[p_{DD}\left(y_{d}|x,y_{d}<k+1\right)\right]\\ \; & -E_{x_{f}∼p_{G}\left(G\left(x_{f}|\left(x,y_{g},y_{d}\right)\right)\right)}\log\left[p_{DD}\left(y_{d}=k+1|x_{f}\right)\right]\\ \; & +L_{T}, \end{array}$$

where $y_{g}$ for gesture type, $y_{d}$ for domain (subject id), *k*—domain categories.

The generator objective function $L_{G}$ consists of three loss terms. The first and second terms motivate the opposite of the discriminator, as described in $L_{D}$. The first term corresponds to the domain discriminator (DD) with the fake sample ($x_{f}$, $y_{d}$) as input, where the domain (subject ID) is $y_{d}<k+1$. This ensures that the generator is penalized if its generated sample $x_{f}$ is classified by the domain discriminator as an unknown (k+1)th domain category. The second term defines the gesture discriminator with ($x_{f}$, $y_{g}$) as the input, where $y_{g}$ is the corresponding gesture category. Finally, the final loss term is the $L_{1}$ loss between the original CSI amplitude *x* and fake amplitude $x_{f}$, weighted with constant β.

(2) $$\begin{array}{cc} L_{G}= & -E_{x_{f}∼p_{G}\left(G\left(x_{f}|\left(x,y_{g},y_{d}\right)\right)\right)}\log\left[p_{DD}\left(y_{d}|x_{f},y_{d}<k+1\right)\right]\\ \; & -E_{x_{f}∼p_{G}\left(G\left(x_{f}|\left(x,y_{g},y_{d}\right)\right)\right)}\log\left[p_{GD}\left(y_{g}|x_{f}\right)\right]\\ \; & +\beta [E_{x∼p_{data}\left(x\right),x_{f}∼p_{G}\left(G\left(x|\left(y_{g},y_{d}\right)\right)\right)}||x-x_{f}|{|}_{1}], \end{array}$$

where $y_{g}$ for gesture type, $y_{d}$ for domain (subject id), *k*—domain categories.

## 5. Performance Evaluation

### 5.1. WiFi CSI Dataset and Computing Resources

**Figure 8:**
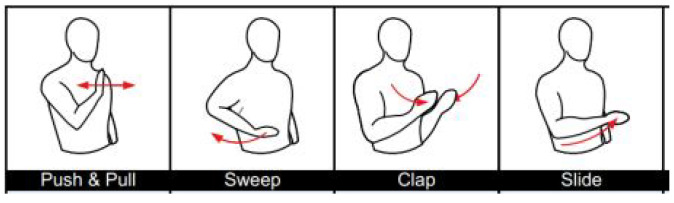
Widar3.0 dataset gesture samples, taken from [5].

**Figure 9:**
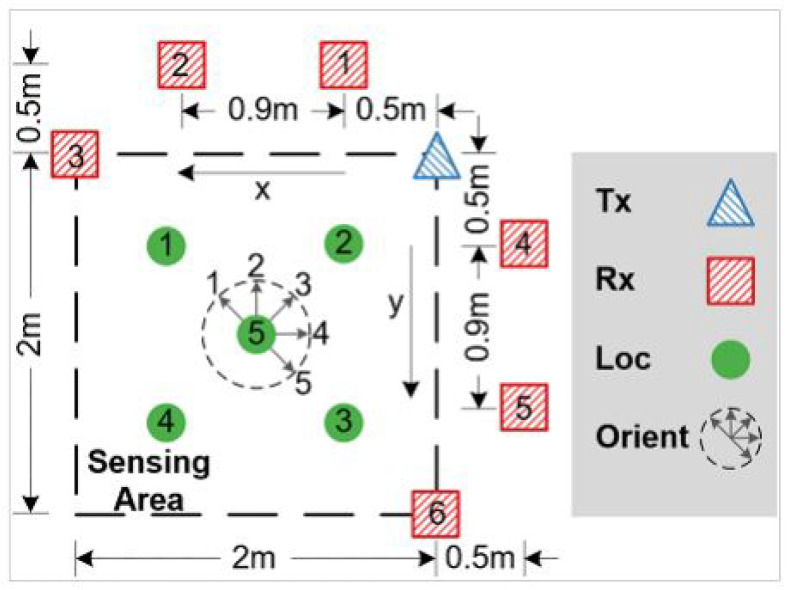
Experiment setup of Widar3.0 dataset, taken from [5].

We used a large and rich open-source WiFi CSI dataset called Widar3.0 [5], which has been used by many other researchers for model training and performance evaluation. The dataset contains WiFi CSI data collected from 17 people performing different hand gestures such as push-pull, sweep, and clap, (see Figure 8 for some example gestures) in three different environments, i.e., classroom, hall and office (identified as room 1, room 2, and room 3, respectively). Experiments were performed at each room, following the setup depicted in Figure 9, consisting of one transmitter and six receivers placed in different positions. Each human subject had to perform each gesture at five different locations and orientations with respect to the transmitter. Correctly recognizing gestures is beneficial for applications in sign language, simultaneous translation for people with hearing difficulties, dementia, gaming, etc, where gestures performed by users need to automatically be recognized by a computer/machine.

**Figure 10:**
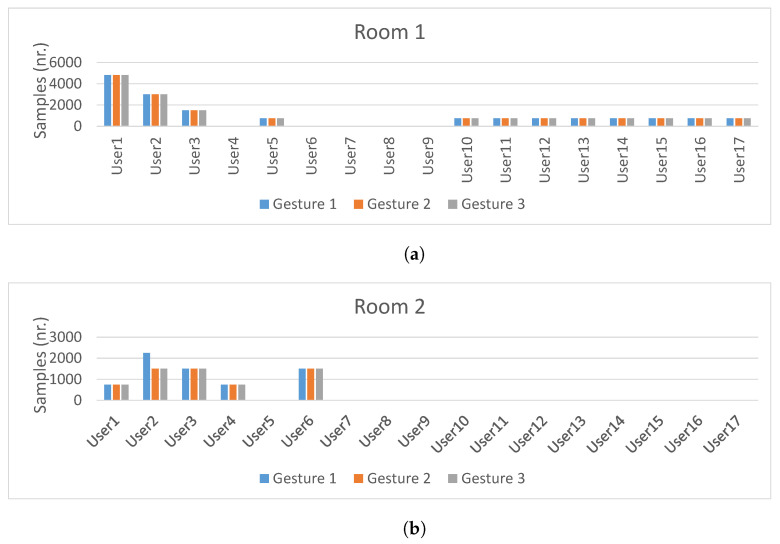

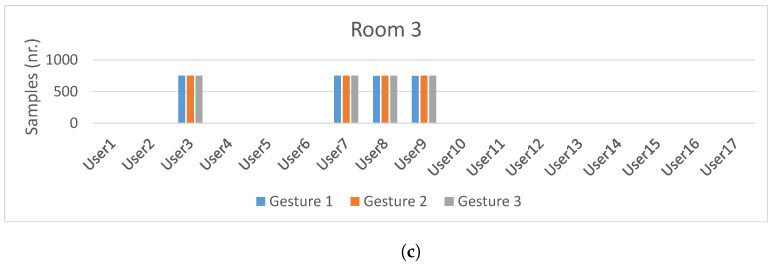
Data distribution of Widar3.0 dataset per subject in different rooms, taken from [5]. (**a**) Subjects in room 1; (**b**) Subjects in room 2; (**c**) Subjects in room 3.

Data sample distribution per subject in each room is depicted in Figure 10. As it can be seen, the largest amount of data samples were collected by subjects 1, 2 and 3. The other subjects collected approximately 2000 CSI samples each. Furthermore, the data set is gesture class balanced: an approximately equal number of the three gesture types were performed by each subject in each room. Overall, this data set will be used for our experiments in the following section, as it allows us to study the impact of the domain change problem caused by change of rooms, change of human subjects, and change of user orientation and location with respect to a transmitter.

To train and test our approaches presented in Section 4.3 and Section 5.3.1, using the Widar3.0 dataset, we used, in terms of computing infrastructure and resources, a Linux server (Ubuntu 18.04.5) with Intel(R) Core(TM) i7-6900K CPU (3.20 GHz) and 126 GB RAM that has two NVIDIA “TITAN X” graphic processing units with 12GB of video RAM each. The simulations were done in Python and we used Tensorflow and Keras frameworks for building and training the models.

### 5.2. Data Pre-Processing

To analyze the effect of various pre-processing steps on the model performance, we used part of the Widar3.0 dataset, as represented in Table 1, for experiments. Two people, with the user IDs’ 1 and 2, were taken for experiments for the training set and test set, respectively. Users performed three hand gestures: push/pull, sweep and clap (Figure 8), in room 1 (classroom) and location 5 (Figure 9).

#### 5.2.1. Effect of Interpolation

**Figure 11:**
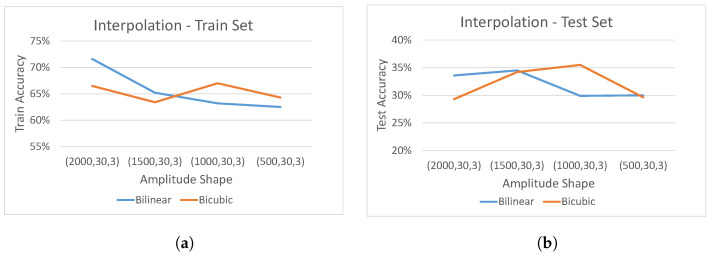
Effect of interpolation with varying input size *N*. (**a**) Model train accuracy. (**b**) Model test accuracy.

As the CSI sample shape is similar to a digital image with three channels [Height,Width,3], image processing interpolation methods were used in accordance with two survey papers, i.e., [30,31]. These survey papers indicate that bi-linear and bi-cubic interpolation methods are computationally cheap, having relatively low peak signal to noise ration. Therefore, we study the impact of bi-linear and bi-cubic interpolation (without normalization step) on the accuracy of our convolutional neural network trained over 11 epochs with varying input size [N, 30, 3], when *N* decreases from 2000 to 500. As can be seen from Figure 11a, while the bi-linear interpolation training accuracy gradually decreases, the accuracy of the bi-cubic interpolation remains approximately the same across all varying values of *N*. This indicates that bi-cubic interpolation is more robust against varying resolution of input data and, therefore, will be used for the final pre-processing module. Regarding the test set accuracy, as can be seen in Figure 11b, we observe that the model performed almost like random guessing, when both interpolation methods were used, with bi-cubic performing slightly better for lower values of *N*.

#### 5.2.2. Effect of Normalization and DWT De-Noising

**Figure 12:**
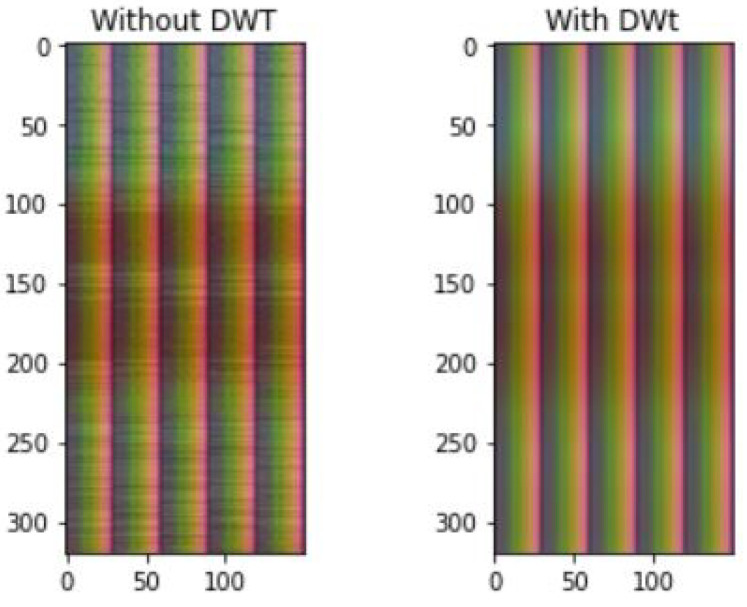
Amplitude sample visualized with and without DWT (wavelet: “sym3”, decomposition threshold 0.5).

**Figure 13:**
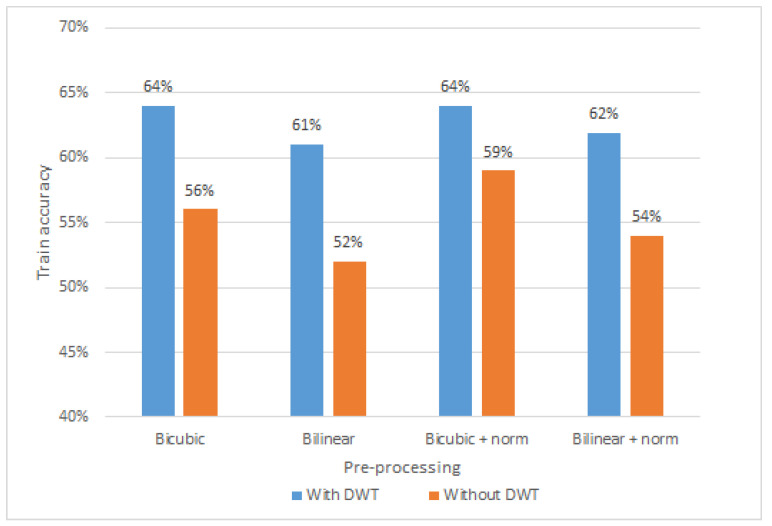
Discrete Wavelet Transform and batch normalization pre-processing results.

In this section, the impact of batch normalization and Discrete Wavelet Transform (DWT) de-noising on the training accuracy of our convolutional neural network model is studied. Firstly, de-noising helps to remove high frequency components from input data, which do not contribute to model performance. In particular, Figure 12 depicts the comparison of amplitude sample with and without Discrete Wavelet Transform. The amplitude sample is first pre-processed with bi-cubic interpolation (shape [1600,30,3]), then reshaped for visualization purposes (shape [320,150,3]). As the final step, DWT is applied, resulting in smoothed shades and colors. As it can be seen in Figure 13, four different pre-processing configurations of bi-cubic interpolation with and without DWT de-noising are compared, when CNN model was trained over 11 epochs. In particular, we experiment with (i) bi-cubic interpolation, (ii) bi-linear interpolation, (iii) bi-cubic with batch normalization, and (iv) bi-linear with batch normalization. First of all, it can be observed, that average training accuracy (62.75%) of all configurations with DWT is higher than without it by approximately 7.5%. This shows that DWT de-noising of input data contributes to a better model training process, leading to a higher recognition accuracy than without it. Although, DWT shows positive results on model training accuracy, it will not be further used in this paper, due to the fact that, our main objective, as it was already mentioned, is to simplify the pre-processing pipeline by finding minimal pre-processing steps that convolutional neural network could work with.

Additionally, we noticed that training a model with batch normalization showed higher training accuracy than without. It can be seen in Figure 13 that the models trained with bi-cubic interpolation and bi-linear interpolation with batch normalization show approximately 2 to 3 percent higher training accuracy. This indicates that normalization of the input data contributes to a better model performance, and therefore the pre-processing pipeline with bi-cubic interpolation and batch normalization will be used for further analysis in Section 5.3 and Section 5.4.

### 5.3. CNN-Based Feature Extraction Hyper-Parameter Study

In this section we focus on the deep learning model used in the feature extraction part of the pipeline. In the previous Section 5.2, we used a simple 4-layer CNN as a feature extractor to identify the minimal pre-processing operations that a deep learning model could work with and still achieve an accepted activity recognition accuracy on the training data set. However, based on the experimental results (Figure 11), we observed that the CNN model used was not robust in the presence of domain change—change of user, when the model was trained on one person and validated on another. Thus, in this section we re-use the data pre-processing module found and propose a different model for feature extraction. In particular, we improve the Convolutional Neural Network model and its objective function in order to address the domain change problem. To analyze theeffect of different parameters and design choices of our CNN model on the accuracy of activity recognition in the presence of domain change, we use part of Widar3.0 dataset, as represented in Table 2. The CNN model takes all gesture samples performed by all subjects in room 1, except subject 11, which is used only for testing purposes.

#### 5.3.1. Training and Testing CNN Model

Regarding the training configuration, the network showed in Figure 5 weights were initialized with Glorot uniform distribution [27] and trained using the Adam [28] optimizer, with a learning rate of $10^{-3}$ for all experiments. Since the task is to classify person gestures, i.e., push/pull, sweep, and clap, we used a combination of cross-entropy $L_{c}$ and triplet loss $L_{T}$, with margin α=1 (originally coined by [32]) as an objective loss function $L=L_{c}+L_{T}$ and aim to minimize it. By adding triplet loss in the objective function, we sought to improve model training convergence, by forcing it to group CSI gesture samples into clusters in the embedding space, with *z* dimensions (a.k.a. latent dimension). This will be discussed in more detail in Section 5.3.4. For model training, we used data that creates conditions of domain change issue. For that we use samples from all users in room 1 except user 11, which was used only for testing purposes. The samples include all gesture samples collected at all locations and face orientations with respect to the transmitter. Due to the highly time consuming training procedure, only the first three user gesture repetitions were used in training and test datasets.

#### 5.3.2. Effect of Max Pooling and Kernel Stride

**Figure 14:**
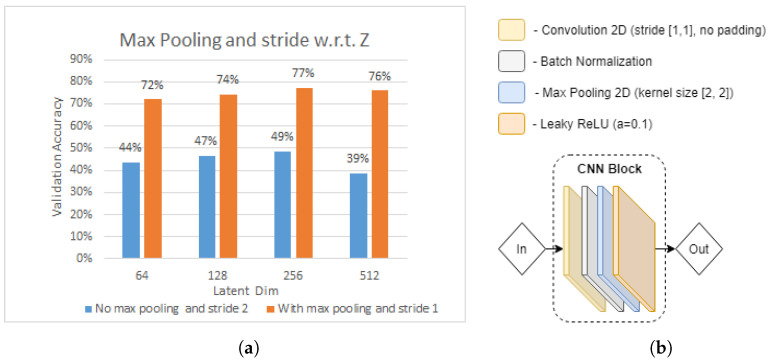
Effect of adding max pooling and changing convolution kernel stride. (**a**) Test accuracy of CNN feature extractor max-pooling and stride w.r.t latent dimension *z* experiment. (**b**) Feature extractor CNN block.

In the very first experiment, we strived to analyse the effect of changing CNN block parameters of the base model in Figure 5. As can be seen in Figure 5, the base model CNN block consists of a convolution operation, followed by batch normalization and LeakyReLU (a=0.1). Then, as depicted in Figure 14b, we alter the CNN block, by inserting max pooling operation and changing the convolution kernel stride to 1. Based on [25], the max pooling layer allows the extraction of statistics that are invariant to small changes of the input to the convolutional layer. This may help to address the shortcomings of the minimal pre-processing, which were found not to be contributing to addressing the domain change issue.

Figure 14a illustrates the results of our CNN model with and without max pooling in terms of validation accuracy, with respect to latent dimension *z* of the densely connected layer in the feature extractor module. As the first observation, we noticed that the CNN model with max pooling and convolution kernel stride 1 outperforms the CNN model without max pooling and convolution kernel stride 2 by, on average, approximately 30%. This shows that the former has a high impact on model performance when tested on a “not seen” user. Moreover, the impact of latent dimension *z* can be identified. We observe that model test accuracy positively correlates with the increasing latent dimension *z*, from 64 to 512 in both CNN block types, with the test accuracy peaking at z=256. As a result, based on this experiment, we further use z=256 and modified version of CNN block, depicted in Figure 14b.

#### 5.3.3. Effect of Dropout Regularization

**Figure 15:**
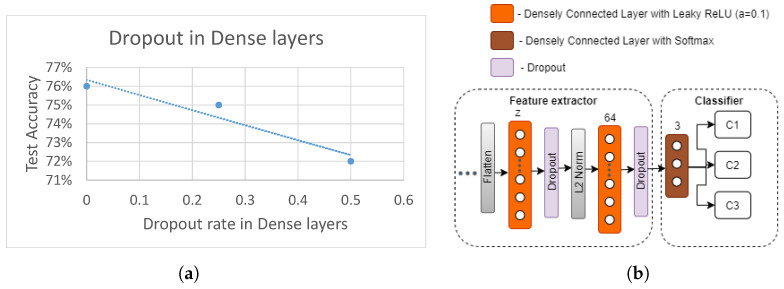
Effect of dropout regularization on test accuracy. (**a**) Dropout regularization results. (**b**) Classifier module with dropout regularization.

In this section, we evaluate the effect of adding the dropout regularization to parts of the feature extractor module (Figure 5). Based on the previous experiment (Figure 15a), the best CNN architecture was chosen and, as represented in Figure 15b, a dropout layer was added after each densely connected layer. The test accuracy of the resulting new model with varying dropout probabilities *p* are shown in Figure 15a. It can be observed that test accuracy negatively correlates with increasing *p*, showing the best test accuracy when p=0. This indicates that dropout regularization after each densely connected layer in the feature extractor is not contributing towards better accuracy, when user 11 is left out of testing. Therefore, dropout regularization is not used in further experiments in this section.

#### 5.3.4. Effect of Triplet Loss Function

The triplet loss function aims to group CSI gesture samples into three clusters (3 categories) in the embedding space of *z* dimensions. Based on [32], this is achieved by maximizing the L2 distance between the samples belonging to different categories with a margin of at least α and minimizing the distance samples belonging to the same category. As depicted in Figure 5, the embedding of each gesture sample is computed by passing it through the CNN module, followed by a densely connected layer, with *z* neurons, and $L_{2}$ normalization.

**Figure 16:**
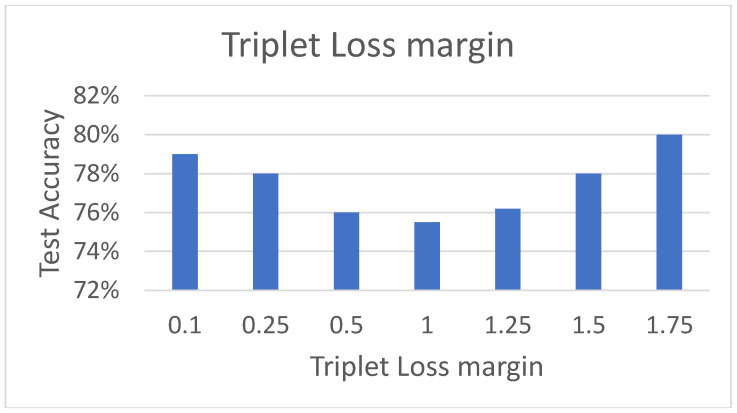
Effect of triplet loss margin.

Based on the previous experiments, we take the best model architecture, which contains max pooling operations in each CNN block and embeds gesture samples into size z=256 vector (embedding space). Figure 16 shows test accuracy with varying α. It can be seen that increasing the margin α from 0.1 to 1 results in decreasing test accuracy, while, on the other hand, increasing α from 1 to 1.75 shows an increasing trend, peaking at 80%, when α=1.75. This indicates that triplet loss with sufficiently high margin contributes to addressing the domain change problem. Thus, a margin of α=1.75 is used for further experimentation in the following Section 5.4.

**Figure 17:**
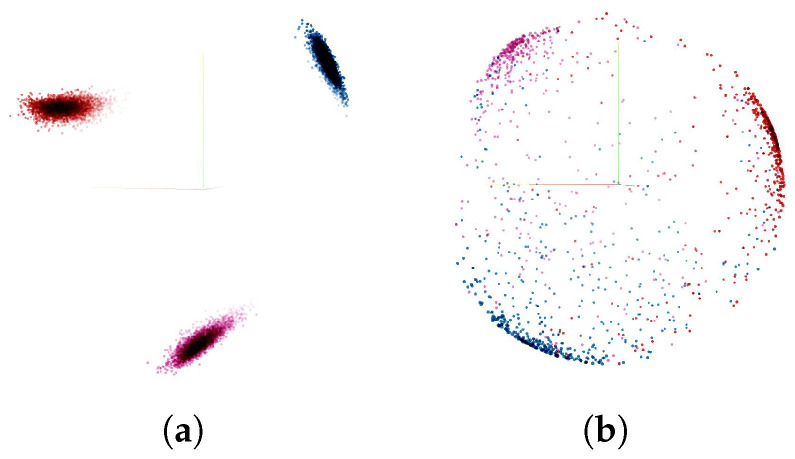
Visualization of effect of triplet loss. Each cluster corresponds to a gesture type. (**a**) Training set embedding vectors visualized in 3-dimensional space with PCA. (**b**) Test set embedding vectors visualized in 3-dimensional space with PCA.

We further analyze the effect of the triplet loss by visualizing the embedding vectors. Firstly, we take the trained model with triplet loss margin of α=1.75 to compute embedding vectors of each gesture sample in train data set and test data set. Then each embedding sample vector of size z=256 was reduced to the size of z=3, using Principal Component Analysis (PCA) for visualization purposes in three dimensional space. The resulting visualization can be seen in Figure 17. It can be observed that three clusters corresponding to each gesture category were obtained. While in the training set, no outliers were observed (see Figure 17a), forming almost perfect clusters. The test set showed clusters, which were quite scattered and had a number of outliers (see Figure 17b). This confirms the lower test accuracy (80%) compared with the training accuracy (99.99%), indicating worse model performance in the presence of domain change.

Overall, triplet loss forces the model to automatically group all gesture samples into clusters in the embedding space, regardless of where, when, and who from, the gesture samples were taken. By doing so, it contributes to addressing the domain change problem.

### 5.4. Adversarial Domain Adaptation

Results described in Section 5.3 show that a certain degree of robustness in the domains of different subjects and rooms was achieved by fine tuning different hyper-parameters of the feature extractor CNN model. In this Section, we further seek to improve model performance in the presence of domain change by utilizing domain information and adversarial training. To study the effect of different design choices of our adversarial network architecture on the accuracy of activity recognition in the presence of domain change, we performed a number of experiments. In particular, we studied the effects of the $L_{1}$ loss term in Section 5.4.1, UNet regularization in Section 5.4.2, and Discriminator regularization in Section 5.4.3.

#### 5.4.1. Effect of $L_{1}$ Loss

**Figure 18:**
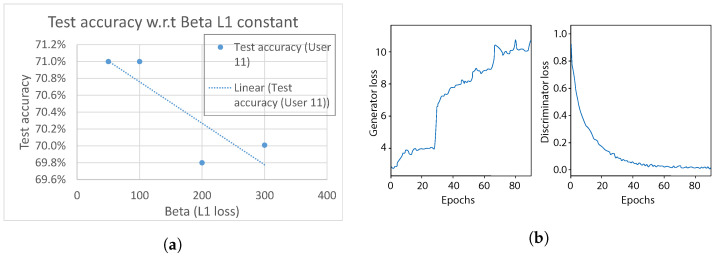
Effect of $L_{1}$ loss. (**a**) Generator $L_{1}$β constant w.r.t test accuracy. (**b**) Comparison between generator and discriminator losses during training, when β=50.

In the first experiment, we analyzed the effect of the $L_{1}$ loss term of the generator, which describes how similar the original input sample *x* and a fake sample $x_{f}$ are. In our model, we vary the constant weight β. As can be seen in Figure 18a, the model test accuracy of gesture classification shows a decreasing trend when β is increased from 50 to 300, peaking at points β=50 & β=100. As the generator aims to translate an input sample *x* in a way that confuses the discriminator, higher values of β force the generator to produce samples that are too similar, resulting in worse performance. Therefore, we select β=50 for further analysis in the following experiments in this Section.

Regarding the domain discriminator classification accuracy, it was observed that it quite quickly reaches approximately 99%, over performing the generator, with loss that showed an increasing trend over the entire training session (Figure 18b). As a consequence, it can be observed that the generator and discriminator are imbalanced. Thus, in order to maintain the balance, we further investigate various regularization methods in the following experiments.

#### 5.4.2. UNet Regularization

In this experiment, we further sought to improve the adversarial training balance between generator and discriminator, by firstly conducting experiments with different regularization methods used in the generator network, which would introduce some “randomness” for fake sample generation. In particular, for each UNet generator layer we tried different regularization methods: dropout, with p=0.25 and addition of Gaussian noise (with standard deviation σ = 0.1 & σ = 0.05). Additionally, we experimented with adding a fourth input, i.e., a Gaussian noise vector of size 256 (σ=0.1) to the generator, and concatenating it with the UNet bottleneck layer the same way as the gesture label, as depicted in Figure 7.

**Figure 19:**
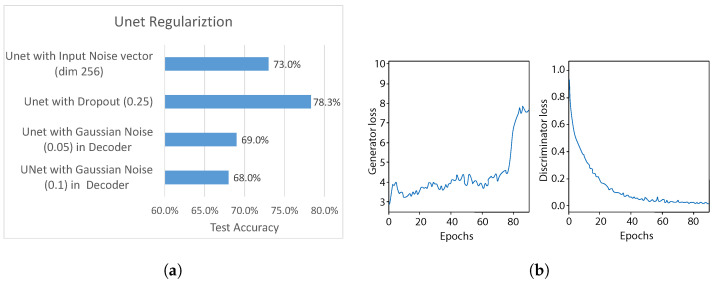
Effect of UNet regularization. (**a**) Experiments of various regularization methods in UNet (generator). (**b**) Comparison between generator and discriminator losses during training, when using dropout in UNet and $L_{1}$ loss β=50.

The test accuracy using each method is shown in Figure 19a. It can be clearly noticed that adding Gaussian noise to each layer output of the UNet decoder part results in the worst performance out of all methods. The standard deviation σ=0.05 shows slightly better results than σ=0.1, reaching approximately 68% and 69% test accuracy. On the other hand, adding (i) Gaussian noise as input to the Unet network and (ii) the dropout, gives stronger results, reaching approximately 73% and 78%, respectively, outperforming the test accuracy of previous experiment. Therefore, we use p=0.25 dropout after each UNet layer as the regularization method for further experiments.

We also analyze the balance between generator and discriminator during training, with the dropout regularization in UNet. As can be seen in Figure 19b, the generator loss, with some fluctuations, stayed at approximately the same value until epoch=60, followed by an increasing trend until the end of the training. This indicates that dropout regularization contributes to improving performance of the generator to compete against the domain discriminator.

#### 5.4.3. Effect of Discriminator Regularization

**Figure 20:**
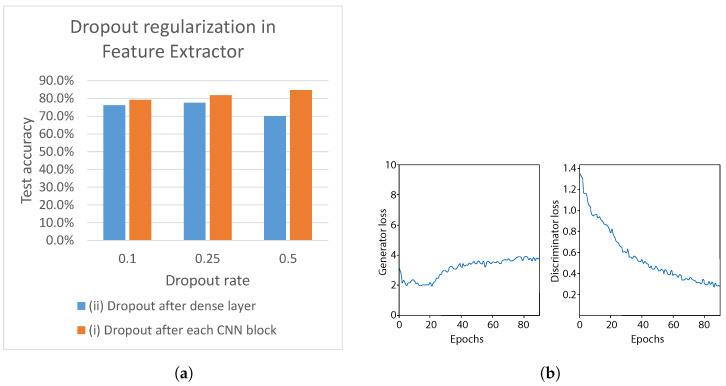
Effect of discriminator regularization. (**a**) Results of applying dropout regularization to the CNN feature extractor and classifier module. (**b**) Comparison between generator and discriminator losses during training, using dropout in UNet and CNN feature extractor, with $L_{1}$ loss β=50.

Experiments in the previous section showed that the dropout positively affected balancing between the generator and the discriminator during the training phase, leading to 78.3% test accuracy. In this section, we further investigate the effect of the dropout on the discriminator network. The effect of dropout regularization with varying dropout probabilities *p* from 0.1 to 0.5 is depicted in Figure 20a. As can be seen, we apply the dropout to different parts of the discriminator network, i.e., (i) after each CNN block of the feature extractor, and (ii) after each densely connected layer in feature extractor (see Figure 6). Applying the dropout to the (i) leads to an increasing accuracy, with test accuracy peaking at 84.7%, when dropout p=0.5. On the other hand, applying the dropout to the (ii) leads to a decreasing accuracy, with the lowest test accuracy 70.1%, when p=0.5. Therefore, we select (i) dropout regularization with p=0.5 for the CNN feature extractor for our final discriminator network.

The training loss plots are shown in Figure 20b. Compared with the previous experiment (Figure 19b), the generator loss shows a more steady trend over 80 epochs of training. Additionally, the discriminator loss shows a decreasing trend, which was not that steep compared with the previous experiment, when no dropout in CNN was used. This indicates that the dropout regularization has a positive effect on balancing the training of the generator and discriminator. As a result, the test accuracy of 84.7% was achieved, which outperforms the best accuracy obtained in Section 5.3.4.

## 6. Discussion

**Figure 21:**
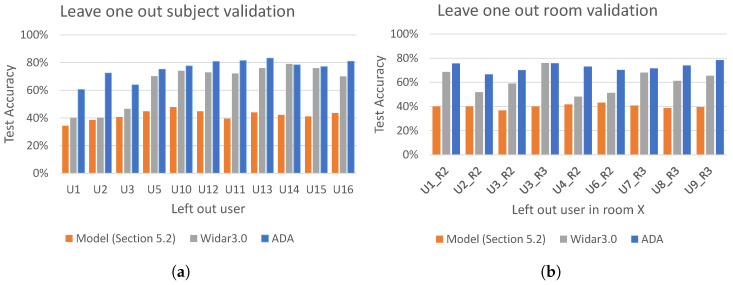
Leave-one-out subject/room validation. (**a**) Leave-one-out subject validation, where x-axis represents left out subject in room 1 for testing. Data set used: room locations—all, face orientations w.r.t Tx—all, gesture sample repetitions—first 3. (**b**) Leave-one-out room validation, where x-axis represents left user in room *x* for testing, e.g., U1_R2—user 1 in room 2. Data set used: room locations—all, face orientations w.r.t Tx—all, gesture sample repetitions—first 3.

Based on the results of the previous experiments, our final generator and discriminator networks look like Figure 6 and Figure 7, with $L_{1}$β=50 (Section 5.4.1), UNet dropout, with p=0.25 (Section 5.4.2) and discriminator, using feature extractor from Section 5.3.4 and dropout, with p=0.5 (Section 5.4.3). Figure 21 shows the overall performance of this model using leave-one-out subject/room validation. We compare the best performing models obtained in Section 5.2, our adversarial domain adaptation network (ADA), and Widar 3.0 [5].

Results of the leave-one-out validation on subject domain are shown in Figure 21a. In each experiment all subjects in room 1 except the one left out were used for training and the left out subject was used for testing. It can be seen that the worst performing model is the one obtained in Section 5.2, with an average test accuracy 41.9%. The Widar 3.0 model achieves an average test accuracy of 65.2%. Finally, regarding the adversarial domain adaptation, it can be seen that its performance is by far the best, showing an average test accuracy of 76%.

The results of leave-one-out room validation on room domain are shown in Figure 21b. In each experiment, CSI gesture samples of all subjects in room 1 were used during training, and CSI samples of one chosen subject, but in different room were used for testing. Similarly as in the leave-one-out subject validation in Figure 21a, the worst and the second worst performing models were the ones experimented in Section 5.2 and Widar3.0, with an average test accuracy of 40.1% and 61%, respectively. Finally, the adversarial domain adaptation shows by far the best performance, with an average test accuracy of 73%.

Overall, the adversarial domain adaptation model shows the highest average test accuracy in the leave-one-out validation for both subject and room domains. Since the domain labels were used as subject ID, the model shows on average better robustness with unseen subjects rather than unseen rooms.

**Figure 22:**
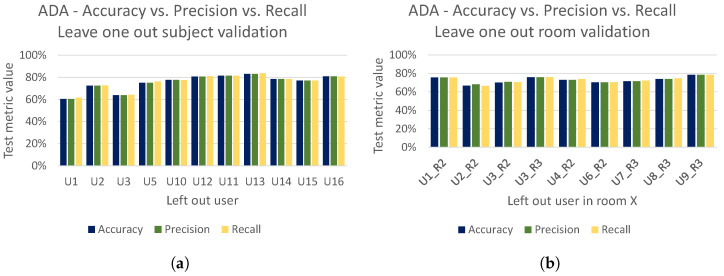
ADA—Accuracy vs. Precision vs. recall. (**a**) Leave-one-out subject validation, where x-axis represents left out subject in room 1 for testing. (**b**) Leave-one-out room validation, where x-axis represents left user in room *x* for testing.

**Figure 23:**
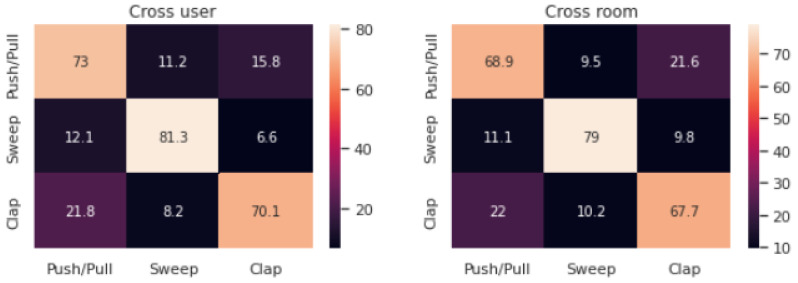
Cross user and cross room confusion matrices of the final ADA model (in % w.r.t. overall class predictions).

To better illustrate the strong performance of our adversarial domain adaptation model (i.e., ADA), we present the accuracy achieved by ADA (the same accuracy as reported in Figure 21a,b) together with the achieved precision and recall for both the leave-one-out subject and room validation in Figure 22a,b, respectively. Finally, in Figure 23 we present cross user and cross room confusion matrices of ADA. Through our extensive evaluations, we have shown that the performance of ADA is consistently high across different domains, and ADA is able to cope well in the context of cross-domain recognition tasks.

## 7. Conclusions and Future Work

We proposed a domain-independent generative adversarial network for WiFi CSI based activity recognition, in combination with a simplified data pre-processing module. We showed that in using this simplified data pre-processing module and utilizing artificially introduced domain shifts via domain-leave-out cross validation, the generative adversarial network outperforms the model presented in Section 5.2 and the Widar 3.0 model. In addition, the impact of various internal parameters and design choices of the generative adversarial network was analyzed. Overall, various internal parameters and design choices have various impacts, ranging from worsening inference performance, even when there is no domain shift present, to improving inference performance, even in the presence of domain shifts. For example, introducing Gaussian noise vectors to balance the game that the generator and discriminator network are playing does not contribute to eventual better inference performance. Triplet loss, on the other hand, contributes to domain shift effect reduction by forcing gesture samples in respective task class specific clusters in the embedding space.

Unfortunately, besides user, sensor device placement, environment, and user placement with respect to the sensor device, not much is known about the effect of other domain factors inducing domain shifts between data used during training or inference, especially in large scale industrial or social environments. In this regard, future work should be focused on acquiring more datasets, at least in accordance with the quality level set by the Widar3.0 dataset, that include more variety in domain shift inducing factors.

The researchers that have worked on the Widar 3.0 model have shown that, in the presence of latent domains, deep neural network inference performance can be drastically reduced. We hypothesize that this reduction can be lowered by means of incremental learning. In this regard, future work may be focused on how latent domain effects can be observed from input data once a deep neural network has been deployed, and on the creation of domain independent deep neural networks that provide support for latent domains.

We observed that models that use domain labels for learning extraction of domain independent features do not scale well to increases in domains, because they require new discriminator construction. Future work could be focused on two different paths. The first path involves developing deep neural network modules that learn to extract domain independent features without a domain label. Existing examples include an attention layer [33] or few-shot learning [34]. The second path involves finding hyperparameter optimization algorithms that have a time complexity that fits within the bounds of training a single deep neural network, and combining this with a reinforcement learning task where an agent decides on domain labeling samples and constructing a multi-task domain label classification problem. The hypothesis here is that, when the model heads are taken off, and a task of interest head is placed on top, the backbone weights already prioritize cross-domain feature extraction.

In this paper, we focused on scenarios in which multiple subjects can be present in a single space and each subject performs an activity/gesture at a time. Future work may consider scenarios in which multiple people at a single space perform the same activity/gesture simultaneously. This, however, requires the collection of appropriate datasets to capture this scenario, since there is no such data available at present.

## Figures and Tables

**Table 1:** Part of Widar3.0 dataset (described in Section 5.1) for pre-processing pipeline experiments.

Number of Categories	Train Set	Test Set	Room Location	Room Type	Face Orientation w.r.t Tx	Amplitude
3	User ID 1	User ID 2	5	Room 1	All	+

**Table 2:** Widar3.0 dataset, discussed in Section 5.1.

Number of Categories	Train Set	Test Set	Room Location	Room Type	Face Orientation w.r.t Tx	Repetition ID
3	U1, U2, U3, U5, U10, U12, U13, U14, U15, U16, U17	User ID 11	All	Room 1	All	1–3

## Data Availability

The Widar3.0 dataset can be accessed via IEEE DataPort here.
